# Constitutive Characterization of FeCoCrNi High-Entropy Alloy During Thermomechanical Deformation Using a New Zerilli–Armstrong Model

**DOI:** 10.3390/ma19132716

**Published:** 2026-06-24

**Authors:** Ali Abd El-Aty, Abdallah Shokry, Mohamed M. Z. Ahmed, Arafa S. Sobh

**Affiliations:** 1 Department of Mechanical Engineering, College of Engineering at Al Kharj, Prince Sattam Bin Abdulaziz University, Al Kharj 11942, Saudi Arabia; 2 Department of Mechanical Engineering, Faculty of Engineering, Fayoum University, Fayoum 63514, Egypt; 3 Department of Mechanical Engineering, Faculty of Engineering, Capital University (Formerly Hewan University), Cairo 11795, Egypt

**Keywords:** constitutive model, hot deformation, Zerilli–Armstrong, FeCoCrNi, high-entropy alloys, sustainability

## Abstract

The thermomechanical deformation behavior of high-entropy alloys (HEAs) is governed by complex interactions among strain, strain rate, and deformation temperature, necessitating robust constitutive models for accurate flow stress prediction and process optimization. In this study, a novel Zerilli–Armstrong (NZA) constitutive model was developed to characterize the hot deformation behavior of FeCoCrNi HEA. The proposed NZA model incorporates enhanced descriptions of strain hardening and deformation-temperature coupling to improve prediction accuracy. The predictability of the proposed NZA model was systematically evaluated and compared with that of the original Zerilli–Armstrong (ZA) and modified Zerilli–Armstrong (MZA) models using key statistical indicators, including the correlation coefficient (R), average absolute relative error (AARE), and root mean square error (RMSE). The findings demonstrate that the NZA model exhibits superior predictive performance, achieving an excellent correlation coefficient (R) of 0.997, a low AARE of 4.22%, and an RMSE of 5.82 MPa. These results confirm the reliability and effectiveness of the proposed constitutive framework in accurately describing the thermomechanical flow behavior of FeCoCrNi HEA over a wide range of deformation conditions. The proposed NZA model provides a robust framework for optimizing hot-forming processes and improving the manufacturing performance of HEA-based components while promoting sustainable manufacturing through reduced material consumption, enhanced energy efficiency, and support for SDGs 9 and 12.

## 1. Introduction

High-entropy alloys (HEAs) represent a groundbreaking advancement in materials science, characterized by their unique composition of five or more principal elements in near-equiatomic ratios, rather than relying on a single base element as in conventional alloys. This innovative concept was first introduced by Yeh et al. [1]. HEAs demonstrate exceptional performance under high-temperature conditions, with their multi-element nature endowing them with superior thermal stability, remarkable mechanical strength retention, and enhanced resistance to oxidation, creep, and thermal softening compared to traditional alloys [2,3]. These outstanding properties are fundamentally driven by four core physical phenomena: severe lattice distortion, sluggish diffusion kinetics, high configurational entropy, and the synergistic cocktail effect [4]. Consequently, HEAs are ideally suited for extreme-environment applications, including critical components in jet engines, aerospace structures, and nuclear reactors, where conventional superalloys often fail to meet stringent operational demands [5]. However, despite their vast potential, the industrial adoption of HEAs is currently limited by significant processing challenges, particularly concerning their workability and defect formation during large-scale manufacturing [6].

Among various HEA systems, the equiatomic FeCoCrNi family has emerged as particularly promising due to its unique combination of structural characteristics, offering an optimal balance of high strength and ductility across a wide temperature range [7]. Typically exhibiting a single-phase face-centered cubic (FCC) structure, this alloy system also demonstrates excellent corrosion resistance and thermal stability, positioning it as a leading candidate for demanding applications in aerospace and marine engineering [8]. However, the hot deformation behavior of these alloys is highly complex. During thermomechanical processing, such as hot rolling or forging, the material’s response is highly sensitive to processing parameters, including temperature, strain rate, and microstructural evolution [9]. The high-temperature deformation of FeCoCrNi HEAs is typically characterized by dynamic softening mechanisms, primarily dynamic recovery (DRV) and dynamic recrystallization (DRX), which compete against work hardening induced by dislocation multiplication [10].

Understanding and accurately modeling hot-flow behavior is essential for optimizing thermomechanical processing conditions and for ensuring the efficient manufacturing of advanced engineering materials. In modern manufacturing, such predictive capability is indispensable for finite element (FE) simulations, which are widely employed to develop processing maps, identify optimal processing windows, prevent flow instabilities such as cracking and localized shear band formation, and predict material performance under demanding service conditions [11,12,13]. Consequently, reliable constitutive models serve as critical tools for process design, manufacturing optimization, and performance assessment. Beyond their scientific and engineering significance, advanced constitutive models play a pivotal role in promoting sustainable manufacturing and accelerating digital transformation [11]. Accurate prediction of material behavior enables virtual process design and optimization, thereby reducing reliance on costly trial-and-error experimentation, minimizing material waste, lowering energy consumption, and shortening product development cycles [9,10,11]. Furthermore, robust constitutive descriptions facilitate the implementation of intelligent manufacturing systems, digital twins, and Integrated Computational Materials Engineering (ICME) frameworks, which are fundamental to next-generation resource-efficient production technologies [12]. By improving process predictability, enhancing resource utilization, and reducing environmental impacts, the development of reliable constitutive models contributes directly to sustainable development goals (SDGs) such as SDG 9 (Industry, Innovation and Infrastructure) and SDG 12 (Responsible Consumption and Production), supporting the transition toward sustainable, efficient, and environmentally responsible manufacturing systems [14].

The constitutive modeling of hot deformation behavior generally involves three principal approaches. The first comprises physically based models founded on fundamental deformation mechanisms, such as dislocation dynamics and recrystallization [15,16,17]. The second includes phenomenological models that establish empirical relationships between flow stress and processing parameters for practical engineering applications [18,19,20,21]. The third involves machine learning-based models, which employ advanced computational techniques to capture complex nonlinear relationships within large datasets [22,23,24]. While phenomenological models like the Johnson–Cook or Arrhenius-type equations are widely used due to their simplicity and ease of integration into FE software, they often lack insight into the underlying physical mechanisms. They may fail to accurately capture flow stress variations across wide ranges of strain rates and temperatures [18,19,20,21]. Conversely, physics-based approaches provide a more robust framework for capturing the true material response across varying conditions by directly linking macroscopic flow behavior to microscopic deformation mechanisms [22,23,24].

Among physics-based approaches, the Zerilli–Armstrong (ZA) model stands out for predicting hot flow behavior in metals by incorporating thermal activation energy and dislocation dynamics [15]. Originally developed for body-centered cubic (BCC) and face-centered cubic (FCC) metals, the ZA model captures the effects of strain hardening, strain rate, and temperature on flow stress [25,26,27]. However, when applied to complex multi-principal element alloys like HEAs, the original ZA model often falls short. The severe lattice distortion and sluggish diffusion inherent to HEAs alter dislocation mobility and thermal activation volumes, rendering the original ZA model insufficiently accurate for predicting their high-temperature flow behavior [28]. To address these limitations, modified versions of the ZA model have been proposed. For instance, Samantaray et al. [25] improved their accuracy by introducing strain-dependent work-hardening/softening terms and coupling temperature-strain rate effects. Such revisions have successfully adapted the model for various conventional alloys and some titanium-based alloys, demonstrating the evolving sophistication of constitutive modeling [25,26,27,28,29]. Despite these advancements, a robust, highly accurate modified ZA model specifically tailored to the unique thermomechanical response of the FeCoCrNi HEA remains limited in the current literature [25,26,27,28,29,30,31].

To bridge this gap, this work presents a new developed Zerilli–Armstrong (NZA) constitutive model specifically developed to improve the prediction accuracy of hot deformation behavior in the FeCoCrNi high-entropy alloy. The proposed NZA model incorporates complex coupling effects between strain, strain rate, and temperature to better reflect the DRX and hardening mechanisms inherent to HEAs. The NZA model is rigorously evaluated against both the original ZA and another modified ZA model (MZA), with comprehensive performance assessment using statistical metrics including R, AARE, and RMSE. The material parameters for all models were systematically determined by nonlinear regression, using the Levenberg–Marquardt optimization algorithm to minimize residual errors and achieve optimal fit to experimental data. Ultimately, this study aims to provide a reliable predictive tool for the thermomechanical processing of FeCoCrNi HEAs, facilitating accurate FE simulations and supporting their transition from lab-scale to industrial applications.

## 2. Experimental Data

Sajadi et al. [32] conducted a comprehensive investigation of the hot deformation behavior of the FeCoCrNi HEA, examining strain rates ranging from 0.001 to 1 s^−1^ across a wide temperature range of 800 to 1200 °C (in 100 °C increments). Their work provides a detailed characterization of the alloy’s chemical composition, microstructural evolution, and complete stress–strain response under these extreme thermomechanical conditions [32]. Notably, their findings revealed that the activation energy for hot deformation of this alloy is approximately 508 kJ/mol, a process primarily governed by the lattice diffusion of the chromium element. The hot deformation process of the FeCoCrNi HEA exhibits distinct, characteristic stages driven by competing microstructural mechanisms. During the initial stage of deformation, the flow stress increases sharply. This rapid escalation is dominated by strain-hardening effects, in which the generation, multiplication, and entanglement of dislocations significantly increase the internal distortion energy. Following this initial peak, the material response bifurcates depending on the processing conditions. At higher strain rates and lower temperatures, dislocation interaction remains the primary mechanism, leading to sustained high flow stress. Conversely, elevated temperatures and lower strain rates promote thermal softening through enhanced dynamic recovery DRV and DRX processes, leading to a progressive reduction in stress until a steady state is reached. In the FeCoCrNi system, discontinuous dynamic recrystallization (dDRX) occurring along initial grain boundaries acts as the primary restoration phenomenon, facilitated by grain boundary migration and dislocation climb. This dynamic equilibrium between work hardening and dynamic softening aligns closely with findings from both traditional alloys and other medium-to-high entropy systems, as documented extensively in the literature [33,34,35,36,37,38].

For the current analysis, the experimental true stress–strain data from the isothermal hot compression tests conducted by Sajadi et al. [32] were meticulously extracted using PlotDigitizer, a highly reliable computer vision-assisted data extraction software. To ensure the reliability of the digitized data, a statistical evaluation was conducted using PlotDigitizer. A reference dataset was extracted three times independently, each with a fresh calibration of the *x*- and *y*-axes. Comparison with the true reference yielded maximum MSEs of 0.00144 for strain and 0.298 MPa for stress, with maximum percentage errors of 2.40% for strain and 0.65% for stress. Given these low error margins and the conservative extraction procedure, the model is expected to predict true experimental behavior with high confidence. The digitized dataset enabled the development, calibration, and systematic evaluation of three distinct constitutive models. The first is the original ZA model; the second is the modified ZA model (MZA) to account for coupled temperature-strain rate effects; and the third is the newly developed ZA model (NZA), specifically tailored to capture the unique microstructural softening and hardening kinetics of the FeCoCrNi HEA. To ensure optimal fitting to the experimental data, the material constants for all models were determined via nonlinear regression utilizing the robust Levenberg–Marquardt optimization algorithm. Finally, a rigorous comparative analysis was conducted using R, AARE, and RMSE to identify the most accurate and reliable model for predicting the complex flow behavior of the FeCoCrNi HEA across the studied temperature and strain rate regimes.

## 3. Constitutive Modelling

The material constants for each constitutive model described below were determined through an optimization procedure implemented in MATLAB. This computational approach utilizes an iterative nonlinear regression technique based on the Levenberg–Marquardt algorithm [39], which systematically minimizes the sum of squared errors between predicted and experimental stress values. The algorithm operates by treating experimentally measured flow stresses as dependent variables, with strain, strain rate, and temperature as independent predictor variables. Initial parameter estimates are provided to initiate the optimization process, which then converges toward optimal values through successive iterations. As detailed in reference [39], this algorithm combines the Gauss–Newton method and a gradient descent approach to achieve robust parameter estimation, particularly effective for solving nonlinear least-squares problems characteristic of constitutive modeling. The Levenberg–Marquardt algorithm is described by [39]:

(1) $$x_{k+1}=x_{k}-{\left[J^{T}J+\mu I\right]}^{-1}J^{T}\left(z-h\left(x_{k}\right)\right)$$

The model constants are represented by a parameter vector $x={\left[x_{1},\;{\;x}_{2},\;\ldots ,\;x_{t}\right]}^{T}$, where t denotes the number of constants, while experimental stress data are organized into a vector ${\left[z_{1},\;{\;z}_{2},\;\ldots ,\;z_{N}\right]}^{T}$, with N being the total number of measurements. The Levenberg–Marquardt algorithm employs a damping parameter μ and identity matrix I, where setting μ = 0 reduces the method to the Gauss–Newton algorithm. Model-predicted stresses at iteration step k are given by vector $h\left(x_{k}\right)$, and the sensitivity of these predictions to parameter variations is quantified through the Jacobian matrix J (dimensions N×t), defined as:

(2) $$J=\left[\begin{array}{ccc} \frac{{\partial h}_{1}}{{\partial x}_{1}} & \cdots & \frac{{\partial h}_{1}}{{\partial x}_{t}}\\ \vdots & \ddots & \vdots \\ \frac{{\partial h}_{N}}{{\partial x}_{1}} & \cdots & \frac{{\partial h}_{N}}{{\partial x}_{t}} \end{array}\right]$$

This enables systematic optimization by adaptively balancing gradient descent (μ > 0) and quadratic approximation (μ → 0) for robust convergence in the nonlinear regression process.

### 3.1. Zerilli–Armstrong Model (ZA)

The ZA constitutive model, first developed in 1987 [15], established distinct physical formulations for face-centered cubic (FCC) and body-centered cubic (BCC) metallic materials. The original framework proposed two separate model variants to account for the fundamental deformation mechanisms characteristic of each crystal structure. These models can be expressed as follows:

(3) $$\sigma =C_{0}+C_{1}\varepsilon^{0.5}\exp\left(-C_{3}T+C_{4}T\ln\varepsilon^{\cdot }\right)\;\;\text{for FCC}$$

(4) $$\sigma =C_{0}+C_{1}\exp\left(-C_{2}T+C_{3}T\ln\varepsilon^{\cdot }\right)+C_{4}\varepsilon^{n}\;\;\text{for BCC}$$

In the constitutive equations, σ corresponds to the flow stress, ε represents the plastic strain, $\varepsilon^{.}$ signifies the strain rate, and T indicates the deformation temperature, while $C_{0}$ through $C_{4}$ denote material-specific constants that characterize the alloy’s thermomechanical response.

The strain hardening term in Equation (4) serves as the key differentiator between the ZA model formulations for FCC and BCC metals. Although the FeCoCrNi alloy has an FCC crystal structure, both formulations were calibrated against the experimental hot-deformation data of Sajadi et al. [32]. The quantitative comparison revealed that the BCC form (Equation (4)) significantly outperformed the FCC form (Equation (3)), yielding a higher correlation coefficient (R = 0.907 vs. 0.841) and a lower RMSE (31.38 MPa vs. 40.23 MPa). The additive strain-hardening term in the BCC form provides better decoupling of thermal and athermal stress components, which is particularly advantageous for concentrated solid solutions like HEAs, where multiple deformation mechanisms (solid-solution strengthening, lattice distortion, dynamic recrystallization) operate simultaneously. Therefore, Equation (4) was adopted in this study to predict the flow behavior of the FeCoCrNi alloy.

The optimization procedure, initialized with parameter values $x_{0}={\left[185\;210\;0.02\;0.0003-125-0.4\right]}^{T}$ employed the Levenberg–Marquardt algorithm to systematically determine the optimal material constants for the original ZA constitutive model. Through iterative refinement, the algorithm achieved convergence by minimizing the residual sum of squares between experimental stress measurements and model predictions, yielding the final calibrated original ZA model formulation

(5) $$\sigma =12.521+212.92\exp\left(-(-0.0085)\;T+0.0009\;T\ln\varepsilon^{\cdot }\right)-0.009\;\varepsilon^{-3.13}\;$$

### 3.2. Samantaray Modified Zerilli–Armstrong Model (MZA)

Samantaray et al. [25] proposed a modified Zerilli–Armstrong (MZA) constitutive model that significantly enhanced the prediction accuracy of hot deformation behavior in metals. Their key modification introduced a strain-dependent term to account for dynamic work hardening and softening effects, coupled with improved temperature-strain rate interaction parameters. This refined formulation better captures the nonlinear evolution of flow stress during thermomechanical processing, particularly in austenitic steels and nickel-based alloys. Samantaray’s adaptation has been widely adopted for modeling hot-working operations, enabling more reliable flow-stress predictions in industrial process simulations. The model is given by:

(6) $$\sigma =\left(C_{1}+C_{2}\varepsilon^{n}\right)\exp\left[-\left(C_{3}+C_{4}\varepsilon \right)T^{*}+\left(C_{5}+C_{6}T^{*}\right)\ln\varepsilon^{\cdot *}\right]$$

The model incorporates seven material-specific constants ($C_{1}$, $C_{2}$, $n,\;C_{3}$, $C_{4}$, $C_{5}$ and $C_{6}$), where $T^{*}$ represents the temperature difference relative to a reference temperature ($T^{*}=T-T_{r}$). Parameter optimization was performed using the Levenberg–Marquardt algorithm with initial values $x_{0}={\left[170-0.09-3\;0.003\;0.0006\;0.1\;0.0003\right]}^{T}$, which successfully converged to minimize the residual sum of squares between experimental measurements and the MZA model predictions. The final calibrated MZA that was presented by Samantaray et al. [25] takes the following form:

(7) $$\sigma =\left(158.11-0.068\;\varepsilon^{-2.60}\right)\exp\left[-\left(0.005+0.0005\;\varepsilon \right)T^{*}\;+\left(0.093+0.0002\;T^{*}\right)\ln\varepsilon^{\cdot *}\right]$$

### 3.3. The Proposed New Developed Zerilli–Armstrong Model (NZA)

The thermomechanical behavior of the FeCoCrNi high-entropy alloy is highly complex during hot deformation. Samantaray et al. [25] made notable contributions by incorporating coupled temperature–strain and strain rate–temperature interactions into their constitutive model. While this approach has demonstrated improved predictive capability for certain alloy systems [40,41,42], its applicability remains limited to specific compositions [43,44,45]. This limitation stems from the model’s simplified representation of the intricate interdependencies between strain, strain rate, and temperature effects in complex multi-component alloys.

Considering the coupled effects of strain hardening, temperature-induced dynamic recrystallization, and thermal softening, along with the interdependence of strain rate, temperature, and strain rate sensitivity, Equation (6) can be expressed in a more comprehensive form:

(8) $$\sigma =A\left(\varepsilon \right)\exp\left[-B\left(\varepsilon ,\;T^{*}\;\right)T^{*}+C\left(T^{*},\ln\varepsilon^{\cdot *}\;\right)\ln\varepsilon^{\cdot *}\right]\;$$

Under reference conditions (i.e., at the reference strain rate and reference temperature), Equation (8) simplifies to:

(9) σ=A(ε)

**Figure 1:**
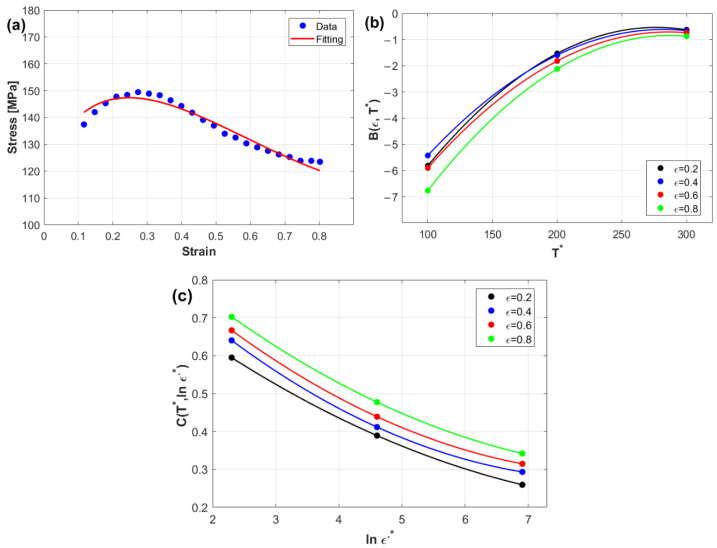
(**a**) stress versus strain, (**b**) $T^{*}$ versus $B\left(\varepsilon ,\;T^{*}\right)$, and (**c**) $\ln\varepsilon^{\cdot *}$ versus $C\left(T^{*},\ln\varepsilon^{\cdot *}\right)$.

The strain hardening term A(ε) is determined by fitting the experimental stress–strain data obtained under reference conditions as depicted in Figure 1a, which enables Equation (9) to be expressed in the following form:

(10) $$\sigma =A_{1}+A_{2}\varepsilon^{\left(A_{3}\varepsilon +A_{4}\varepsilon^{2}\right)}\;$$

When limited to the reference strain rate, the logarithmic transformation and algebraic rearrangement of Equation (8) lead to:

(11) $$B\left(\varepsilon ,\;T^{*}\;\right)=\;\frac{-\ln\left[\sigma /\left(A_{1}+A_{2}\varepsilon^{\left(A_{3}+A_{4}\varepsilon \right)}\;\right)\right]}{T^{*}}$$

The influence of temperature on $B\left(\varepsilon ,\;T^{*}\right)$ is illustrated in Figure 1b. Considering that linear relationships provide only a limited approximation, and that Li et al. [46] demonstrated an exponential relationship between activation volume and temperature in high- and medium-entropy alloys of the Cr-Mn-Fe-Co-Ni system, along with the effect of temperature on strain rate sensitivity (Figure 1b), the relationship between temperature and strain rate sensitivity can be represented as a polynomial. Following Samantaray et al. [25], where strain is treated as a linear function when coupled with temperature, $B\left(\varepsilon ,\;T^{*}\right)$ can be expressed as the sum of a linear strain term and a polynomial temperature function. This leads to the following proposed formulation:

(12) $$B\left(\varepsilon ,\;T^{*}\right)=B_{1}+B_{2}\;\varepsilon +B_{3}\;T^{*2}\;$$

At reference temperature, taking logarithms and rearranging Equation (8) gives

(13) $$C\left(T^{*},\ln\varepsilon^{\cdot *}\right)=\frac{\ln\left[\sigma /\left(A_{1}+A_{2}\varepsilon^{\left(A_{3}+A_{4}\varepsilon \right)}\;\right)\right]}{\ln\varepsilon^{\cdot *}}$$

The strain rate dependence of $C\left(T^{*},\ln\varepsilon^{\cdot *}\right)$ is shown in Figure 1c. Linear relationships provide only a limited approximation. As He et al. [47] demonstrated a continuous variation in strain rate sensitivity with strain rate in the FeCoNiCrMn high-entropy alloy, and considering the effect of strain rate on strain rate sensitivity (Figure 1c), the relationship between these two parameters is represented as a polynomial in this work. Following the approach of Samantaray et al. [25], which treats temperature as linearly coupled with strain rate, $C\left(T^{*},\ln\varepsilon^{\cdot *}\right)$ is proposed as a composite function combining a linear temperature term and a polynomial strain rate dependence, leading to the following expression:

(14) $$C\left(T^{*},\ln\varepsilon^{\cdot *}\right)=C_{1}+C_{2}\;\;T^{*}+C_{3}\;{\left(\ln\varepsilon^{\cdot *}\right)}^{2}$$

Consequently, the NZA model based on the discussed modifications that affect strain hardening, dynamic recovery, and softening can be presented as:

(15) $$\sigma =\left(A_{1}+A_{2}\varepsilon^{\left(A_{3}\varepsilon +A_{4}\varepsilon^{2}\right)}\right)\exp\left[-\left(B_{1}+B_{2}\;\varepsilon +B_{3}\;T^{*2}\right)T^{*}\;\;\;\;\;+\left(C_{1}+C_{2}T^{*}+C_{3}\;{\left(\ln\varepsilon^{\cdot *}\right)}^{2}\right)\ln\varepsilon^{\cdot *}\right]$$

In sum, the NZA model introduces three key modifications to enhance prediction accuracy: the strain hardening term is reformulated by expressing the exponent (n) as a polynomial function of strain $\left(A_{3}\varepsilon +A_{4}\varepsilon^{2}\right)$, and thermal effects are more comprehensively captured through a temperature term $\left(B_{3}\;T^{*2}\right)$ that couples both strain and temperature dependencies. Additionally, strain rate sensitivity is improved via an additional parameter $\left(C_{3}\;{\left(\ln\varepsilon^{\cdot *}\right)}^{2}\right)$ that explicitly links temperature and strain rate effects. These modifications collectively provide a more physically realistic representation of the complex interactions among strain accumulation, thermal activation, and rate-dependent deformation mechanisms during hot-working processes. The Levenberg–Marquardt algorithm was applied for parameter optimization, starting with initial values $x_{0}={\left[150-320\;10\;20\;0.007\;0.0006\;3\times 10^{-09}\;0.2\;0.0004-0.002\right]}^{T}$. The algorithm successfully converged, minimizing the residual sum of squares between experimental data and predictions from the MZA model. The final calibrated version of the NZA model is expressed as follows:

(16) $$\sigma =\left(141.13-301\;\varepsilon^{\left(9.12\;\varepsilon +17.78\;\varepsilon^{2}\right)}\right)\exp\left[-\left(0.005+0.0004\;\varepsilon \;+2.07\;\times 10^{-09}\;T^{*2}\right)T^{*}\;\;\;\;\;\;\;\;\;+\left(0.168+0.0003\;T^{*}-0.001{\left(\ln\varepsilon^{\cdot *}\right)}^{2}\right)\ln\varepsilon^{\cdot *}\right]$$

## 4. Results and Discussion

### 4.1. Analysis of Predicted Versus Experimental Stresses

**Figure 2:**
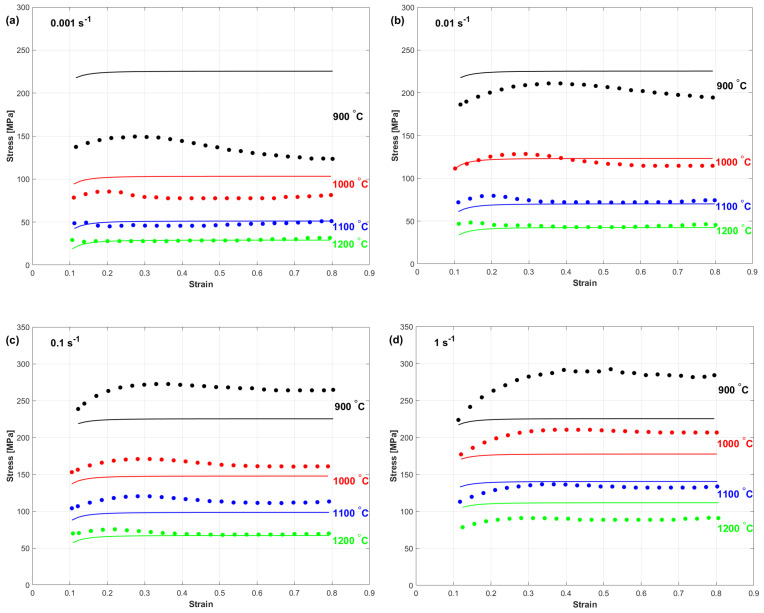
Correlation between experimental (symbols) and original ZA results (lines) at strain rates of (**a**) 0.001 s^−1^, (**b**) 0.01 s^−1^, (**c**) 0.1 s^−1^, and (**d**) 1 s^−1^.

This section presents a detailed comparative analysis of the experimental stress measurements and the predicted stresses generated by three different models: the original ZA, the MZA proposed by Samantaray et al. [25], and the NZA models. The study evaluates the accuracy and reliability of these models in describing the deformation behavior of FeCoCrNi HEA, followed by a critical assessment of their respective strengths and limitations. Figure 2 compares experimental stress measurements with predictions from the original ZA model. The model shows significant deviations across almost all tested strain rates and temperatures Figure 2a–d, indicating its limited ability to describe the flow behavior of FeCoCrNi HEA. These inaccuracies likely stem from the model’s oversimplified treatment of interactions among strain, strain rate, and temperature. Hot deformation involves nonlinear microstructural evolution (e.g., dynamic recovery/recrystallization), which the original ZA formulation fails to capture adequately. This shortcoming becomes especially apparent at elevated temperatures and strain rates, where the model’s predictions diverge markedly from experimental data. Consequently, the original ZA model is insufficient for reliable stress prediction in FeCoCrNi HEA, underscoring the need for revised formulations that better account for coupled thermo-mechanical effects.

**Figure 3:**
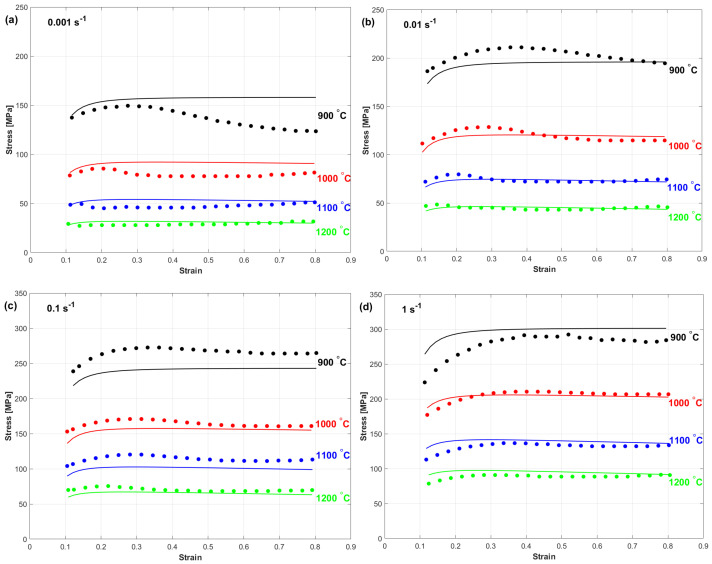
Correlation between experimental (symbols) and MZA model results presented by Samantaray et al. [25] (lines) at strain rates of (**a**) 0.001 s^−1^, (**b**) 0.01 s^−1^, (**c**) 0.1 s^−1^, and (**d**) 1 s^−1^.

Figure 3 compares experimental stress measurements with predictions from the MZA model proposed by Samantaray et al. [25]. While the MZA model shows improved accuracy over the original ZA formulation, it still exhibits notable deviations under specific conditions: at 900 °C (all strain rates), 1000 °C (0.001 s^−1^ and 0.1 s^−1^), and 1100 °C (0.1 s^−1^). A clear underprediction is observed at 900 °C across all strain rates. This behavior might be correlated with the incomplete DRX and necklace-like heterogeneous microstructure reported by Sajadi et al. [32] at this temperature. The linear approximations of the MZA model are unable to capture the nonlinear thermal-activation behavior and the mixed microstructure comprising both deformed and recrystallized regions. These inconsistencies reveal persistent limitations in capturing the complex hot deformation behavior of FeCoCrNi HEA, particularly under varying thermomechanical conditions.

The observed discrepancies likely stem from the inherent challenges in modeling the alloy’s nonlinear flow behavior. Although the MZA model performs well at higher temperatures (1100–1200 °C) and selected strain rates (0.001–1 s^−1^), its simplified constitutive framework struggles to account for dynamic microstructural evolution fully. This shortcoming becomes pronounced under extreme deformation conditions where dynamic recovery and recrystallization dominate, suggesting the need for a more sophisticated representation of temperature–strain rate–strain interdependencies.

Notably, the MZA model demonstrates reasonable predictive capability for certain temperature–strain rate combinations, attributable to its improved treatment of strain-rate interactions. This represents a clear advancement over the original ZA model. However, its inability to consistently predict flow stresses across all tested conditions underscores the necessity for further refinements, particularly to address the complex deformation mechanisms inherent to FeCoCrNi HEA at intermediate temperatures and strain rates.

**Figure 4:**
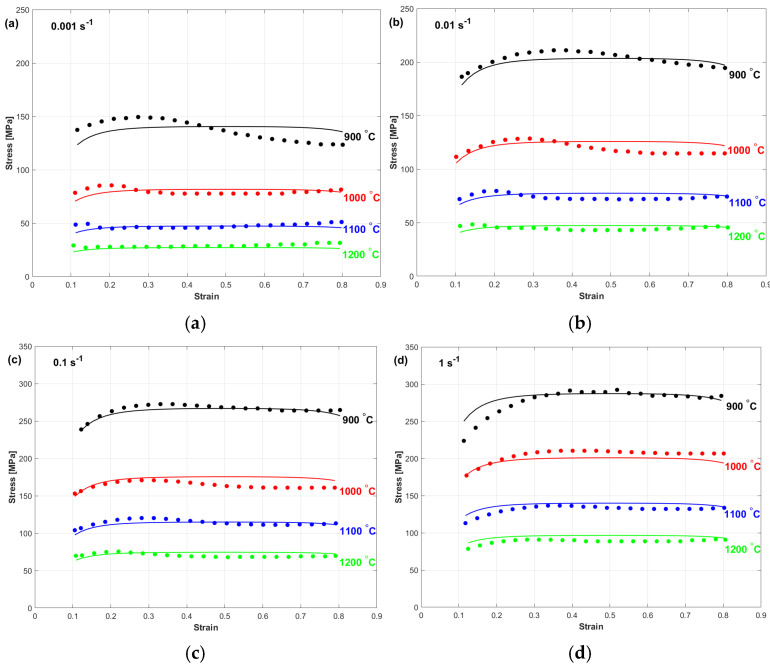
Correlation between experimental (symbols) and the NZA data (lines) at strain rates of (**a**) 0.001 s^−1^, (**b**) 0.01 s^−1^, (**c**) 0.1 s^−1^, and (**d**) 1 s^−1^.

Figure 4 presents a comprehensive evaluation of the NZA model’s performance by comparing its stress predictions with experimental measurements for FeCoCrNi HEA. The results demonstrate superior agreement across all tested temperatures (900–1200 °C) and strain rates (0.001–1 s^−1^), marking a significant improvement over both the original ZA and MZA models. The NZA model shows only a slight underprediction at 900 °C, attributed to its polynomial (quadratic) temperature and strain-rate terms. This minor deviation correlates with the incomplete DRX and necklace-like heterogeneous microstructure reported by Sajadi et al. [32] at this temperature.

This consistent accuracy confirms the NZA model’s enhanced capability to capture the alloy’s deformation behavior under diverse thermomechanical conditions. The model’s exceptional performance stems from its improved treatment of strain–strain rate–temperature coupling effects. Unlike its predecessors, the NZA model successfully integrates these interdependent factors, enabling more accurate simulation of the alloy’s nonlinear flow behavior. This advancement is particularly crucial for FeCoCrNi HEA, where concurrent microstructural phenomena—including work hardening, dynamic recovery, and recrystallization—collectively govern stress evolution during hot deformation.

These results validate the effectiveness of the NZA model’s modifications in overcoming the fundamental limitations of previous versions. The model’s robust predictive capability across wide processing windows suggests valuable practical applications, from optimizing hot-working parameters to predicting in-service performance of components subjected to elevated temperatures. This represents a meaningful advancement in constitutive modeling for complex alloy systems.

### 4.2. Assessment of Models’ Accuracy

A comprehensive statistical evaluation was conducted to compare the predictive capabilities of the original ZA, MZA, and the proposed NZA models for FeCoCrNi high-entropy alloy. R, AARE, and RMSE [48] were employed to quantify model accuracy across the full range of experimental strain rates and temperatures. R evaluates the linear dependence between experimental and predicted values as listed in Equation (17). R = 1 represents perfect agreement. AARE, as defined in Equation (18), measures the mean absolute percentage difference between measured and calculated stresses and serves as a robust indicator of overall model precision. RMSE, as defined in Equation (19), provides a dimensionless measure of the average prediction error magnitude, with values approaching zero indicating optimal model performance.

(17) $$R=\frac{\sum_{i}^{N}\left(\sigma_{e}-\bar{\sigma_{e}}\right)\left(\sigma_{P}-\bar{\sigma_{P}}\right)}{\sqrt{\sum_{i}^{N}{\left(\sigma_{e}-\bar{\sigma_{e}}\right)}^{2}\sum_{i}^{N}{\left(\sigma_{P}-\bar{\sigma_{P}}\right)}^{2}}}$$

(18) $$AARE=\frac{1}{N}\sum_{i}^{N}\left|\frac{\sigma_{e}-\sigma_{P}}{\sigma_{e}}\right|\times 100$$

(19) $$MSE=\sqrt{\frac{1}{N}\sum_{i}^{N}{\left(\sigma_{e}-\sigma_{P}\right)}^{2}}$$

**Figure 5:**
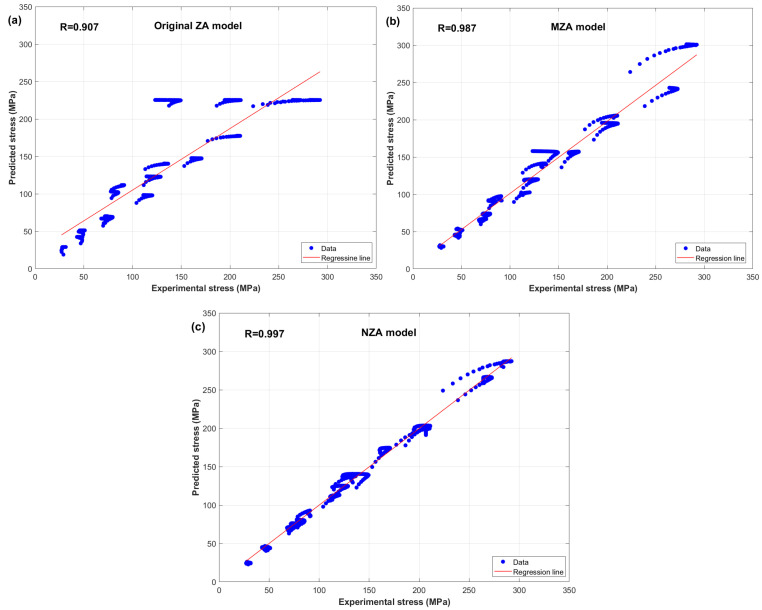
Experimental versus predicted stress correlation for: (**a**) original ZA, (**b**) MZA, and (**c**) NZA models.

In the present analysis: $\sigma_{e}$ signifies experimentally determined stress measurements, with $\bar{\sigma_{e}}$ representing their mean value. The symbol $\sigma_{P}$ corresponds to model-generated stress predictions, while $\bar{\sigma_{P}}$ indicates the mean predicted value. The variable N enumerates the total data points in the dataset. Figure 5 presents a systematic comparison of experimentally measured stresses with those predicted by the original ZA, MZA, and NZA models. The graphical representation enables direct evaluation of each model’s ability to replicate the flow behavior of FeCoCrNi HEA across varied thermomechanical processing conditions. The NZA model demonstrates superior predictive capability, as evidenced by its near-perfect correlation coefficient (R = 0.997) as depicted in Figure 5c. This performance substantially exceeds both the original ZA model (R = 0.907, Figure 5a) and the intermediate MZA model (R = 0.987, Figure 5b). The progressive improvement in R-values across model versions confirms the enhanced capacity of the MZA to capture the alloy’s nonlinear deformation response. The NZA model’s exceptional accuracy stems from its improved treatment of critical deformation phenomena, including strain hardening and dynamic softening mechanisms. These advancements establish the NZA formulation as a robust predictive tool for industrial hot-working process optimization and high-temperature performance assessment of FeCoCrNi HEA.

**Figure 6:**
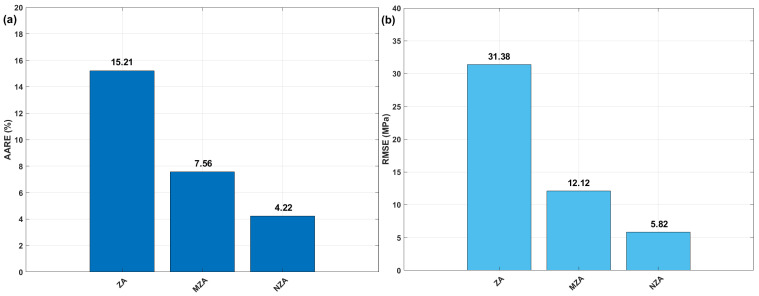
Graphical representation of model performance metrics: (**a**) AARE (%) and (**b**) RMSE (MPa) values for original ZA, MZA, and NZA formulations.

The predictive performance of the original ZA, MZA, and NZA models for FeCoCrNi HEA was quantitatively evaluated using statistical error metrics. Figure 6 presents a comparative visualization of AARE and RMSE across all models. Performance analysis demonstrates substantial improvements in model accuracy through successive modifications. The NZA model achieves exceptional precision with an AARE of 4.22% (Figure 6a), representing a sevenfold improvement over the original ZA model (15.21%). Intermediate performance is observed for the MZA model (7.56%), confirming progressive enhancement. Parallel results are observed in the RMSE values (NZA: 5.82 MPa; MZA: 12.12 MPa; ZA: 31.38 MPa) as depicted in Figure 6b. These quantitative results validate the critical importance of properly accounting for coupled strain-rate–temperature effects in constitutive modeling. The NZA model’s superior accuracy makes it a reliable tool for industrial process optimization, particularly in applications involving dynamic recrystallization.

## 5. Conclusions

In this investigation, a modified Zerilli–Armstrong (NZA) constitutive model was proposed to predict the thermomechanical deformation behavior of the FeCoCrNi HEA. Through a comprehensive statistical analysis using R, AARE, and RMSE, this work rigorously evaluates predictive accuracy across the original ZA, MZA, and the proposed NZA models. The quantitative assessment demonstrates highly significant improvements in accuracy across these iterations. The baseline, original ZA model exhibits severely limited predictive capability for this HEA system (R = 0.907, AARE = 15.21%, RMSE = 31.38 MPa), highlighting the inherent limitations of this model, which assumes a decoupled relationship between strain hardening, strain rate, and temperature. In contrast, the MZA model shows substantial enhancement (R = 0.987, AARE = 7.56%, RMSE = 12.12 MPa) by introducing coupled terms that better approximate high-temperature softening. Ultimately, the proposed NZA model achieves exceptional performance (R = 0.997, AARE = 4.22%, RMSE = 5.82 MPa), representing a remarkable 72% reduction in AARE and 83% in RMSE compared to the original ZA and MZA models. These quantitative improvements directly correlate with the enhanced physical treatment of strain-rate–temperature coupling within the NZA model. While the original ZA model oversimplifies these interactions by treating thermal activation and dislocation glide as independent of accumulated strain, the NZA model successfully captures the highly nonlinear deformation physics governing the FeCoCrNi HEA. By precisely modeling how severe lattice distortion and sluggish diffusion alter dislocation mobility and the thermal activation energy, the NZA model accurately predicts the evolution of flow stress during both the initial strain-hardening phase and the subsequent dynamic recovery and recrystallization (DRX) softening phases. These theoretical and mathematical advancements establish the NZA model as a highly reliable predictive tool for finite element simulations of industrial thermomechanical processing, with robust statistical evidence confirming its superior accuracy and stability.

## Data Availability

The original contributions presented in the study are included in the article, further inquiries can be directed to the corresponding authors.
